# Application of diffusion tensor imaging technology in glaucoma diagnosis

**DOI:** 10.3389/fnins.2023.1125638

**Published:** 2023-02-02

**Authors:** Jiaqi Wang, Yaqiong Zhang, Xueyu Meng, Gang Liu

**Affiliations:** Department of Ophthalmology, Xiangyang No. 1 People’s Hospital, Hubei University of Medicine, Xiangyang, Hubei, China

**Keywords:** glaucoma, diffusion tensor imaging, visual field, OCT, OCTA

## Abstract

Glaucoma is the first major category of irreversible blinding eye illnesses worldwide. Its leading cause is the death of retinal ganglion cells and their axons, which results in the loss of vision. Research indicates that glaucoma affects the optic nerve and the whole visual pathway. It also reveals that degenerative lesions caused by glaucoma can be found outside the visual pathway. Diffusion tensor imaging (DTI) is a magnetic resonance imaging (MRI) technique that can investigate the complete visual system, including alterations in the optic nerve, optic chiasm, optic tract, lateral geniculate nuclear, and optic radiation. In order to provide a more solid foundation for the degenerative characteristics of glaucoma, this paper will discuss the standard diagnostic techniques for glaucoma through a review of the literature, describe the use of DTI technology in glaucoma in humans and animal models, and introduce these techniques. With the advancement of DTI technology and its coupling with artificial intelligence, DTI represents a potential future for MRI technology in glaucoma research.

## 1. Introduction

Glaucoma is a diverse group of diseases characterized by damage to the retinal ganglion cells (RGC) and their axons, the retinal nerve fiber layer (RNFL), which leads to progressive vision loss (Stein et al., 2021). If left untreated, this condition can result in permanent blindness. Glaucoma is thought to affect more than 76 million people worldwide; by 2040, that number is expected to increase to 112 million (Tham et al., 2014). High intraocular pressure (IOP) is a significant risk factor for glaucoma. In some instances, however, reducing the IOP to normal or even below normal can cause visual impairment. IOP is the only clinical risk factor that may be modified. In addition, risk factors for glaucoma include advanced age (Kühn et al., 2021), race (Cheng and Tanna, 2022), myopia (Haarman et al., 2020), and glaucoma-positive family history (Bhandari et al., 2021); nevertheless, the exact reason is unknown (Schuster et al., 2020). Epidemiology studies show that the pathophysiological pathways of glaucoma are similar to those of Alzheimer’s disease and Parkinson’s disease. They suggested that the brain may be involved in the development of glaucoma (Zhang et al., 2019; Saccà et al., 2020). Historically, glaucoma referred to illnesses resulting in optic nerve (ON) atrophy and visual field (VF) abnormalities. However, an increasing amount of evidence indicates that intracranial vision-related regions and visual pathways, such as the lateral geniculate nuclear (LGN), optic tract (OT), and optic radiation (OR), are also affected (Zhou et al., 2017; Schmidt et al., 2018; Song et al., 2018). In addition, some investigations have found a decrease in the volume of brain structures outside the visual pathway (Chen et al., 2013). Visual pathways are made up of white matter (WM) tracts, so studying glaucomatous WM degeneration may be crucial to understanding how glaucomatous neurodegeneration spreads through the visual system.

Diffusion tensor imaging (DTI) is now the most prevalent method for investigating WM degeneration in glaucoma patients (Hanekamp et al., 2021; Haykal et al., 2022). In addition, the DTI technique is frequently utilized to detect glaucoma changes in various glaucoma types (Zhang et al., 2019). In this review, we explored the application of DTI in glaucoma and assessed it as a recently created technique.

## 2. Conventional glaucoma screening methods

Routinely used methods for glaucoma evaluation include gonioscopy, IOP measurement, corneal thickness measurement, fundus photography, VF inspection, and optical coherence tomography (OCT) (Stein et al., 2021). Moreover, optical coherence tomography angiography (OCTA) identifies glaucoma by detecting alterations in the retinal blood vessels (Rao et al., 2020). Due to irreversible glaucomatous vision damage, early detection is essential for preventing its progression. Through screening and identifying high-risk patients, ophthalmologists can diagnose glaucoma in its early stages. Given that the morphology of a healthy ON varies considerably and various eye disorders can present with glaucomatous alterations in the ON, early identification of glaucoma can be difficult. For glaucoma diagnosis, direct visual evaluation of optic papilla morphology has a high mistake rate. Consequently, early glaucoma is identified by longitudinally assessing structural changes in the ocular papilla.

The term “visual field” refers to the area the human eye can perceive, and the “VF examination” primarily refers to the perimeter measuring this region. VF examination is a subjective test used to diagnose glaucomatous damage and quantify the degree and severity of visual impairment in glaucoma patients (Hashimoto et al., 2018). The automated static visual field test effectively detects and monitors visual function loss caused by glaucoma. Even in the earliest phases of glaucoma, more and more macular abnormalities are detected (Arai et al., 2018). Considering the significance of central visual function, some have recently proposed that regular targeted central VF testing, such as the 10-2 approach on Humphrey Field Analyzer (HFA), can detect glaucomatous alterations in their early phases (Grillo et al., 2016). In addition, the 10-2 strategy on the HFA can detect abnormal outcomes in glaucoma patients that the 24-2 strategy cannot detect (Grillo et al., 2016). Therefore, suitable parameter values may facilitate earlier diagnosis of glaucoma. In order to sample the same retinal position, the patient needs to maintain a steady position throughout standard VF testing for SAP. Thus, the researchers utilized a fundus-tracked visual field test technique to lower measurement variability and to be able to quickly detect VF advancement when patient participation declines over time (Wu et al., 2016; Rao et al., 2017). Furthermore, implementing a new visual field threshold technique and an upgraded visual field progression analysis offer promise for the early identification of glaucoma (Aoki et al., 2017; Wild et al., 2017).

Optical coherence tomography is a non-invasive imaging technology that permits the quantitative measurement of changes in the ON, RGC axon, and RGC body layer, RNFL (Kang and Tanna, 2021). Since its inception in 1991 (Huang et al., 1991), OCT has undergone several modifications, including time-domain OCT (TD-OCT), spectral-domain OCT (SD-OCT), and swept-source OCT (SS-OCT) (Geevarghese et al., 2021). These enhancements have substantially increased scanning resolution and speed. OCT has transformed the ability to analyze the anatomical characteristics of an optic papilla afflicted by glaucoma. In addition, OCT can detect glaucoma before VF changes (Swaminathan et al., 2021). OCT has transformed glaucoma from a disease that could only be diagnosed subjectively to one that can now be evaluated objectively. This significant development has revolutionized the diagnosis, monitoring, and treatment of glaucoma. As a result, it has become the most common method for detecting and monitoring glaucoma among clinicians. According to the study, RNFL-based SD-OCT and TD-OCT have the same ability to distinguish glaucoma from other eye diseases. However, SD-OCT is superior for determining RNFL advancement (Bengtsson et al., 2012). Due to the SD-OCT rapid scan speed and excellent images, the retina may be analyzed more precisely, and there is less variance in the results. Finally, it increased the precision of glaucoma diagnosis.

Optical coherence tomography angiography is a non-invasive, dye-free imaging technique that can measure the retina, optic papilla, and choroidal blood vessels in qualitative and quantitative ways. OCTA can provide information on the extent of perfusion damage at various depths in the retina and choroid. Because advanced glaucoma has a lower vascular density (Yarmohammadi et al., 2018), OCTA can be utilized to track the progression of ocular damage in this condition. Patients at risk for a more rapid glaucoma progression are also included in the OCTA database. OCTA works with VF and OCT tests (Rao et al., 2020) to diagnose glaucoma, identify development, and assess progression risk.

Conventional glaucoma diagnostic technologies, including fundus photography, OCT, OCTA, and VF, focus exclusively on the retinal region and disregard information regarding the WM of the brain, which transmits and systematically processes vision. The visual pathway consists mainly of the WM of the brain, and DTI is the advanced imaging technology currently available for evaluating the WM of the brain (Cheng et al., 2021); consequently, DTI is essential for detecting glaucoma.

## 3. Application of DTI technology in glaucoma

Diffusion-weighted imaging (DWI) is a magnetic resonance imaging (MRI) technique used to assess water diffusion (Graham et al., 2021). Due to the presence of water diffusion in numerous types of biological tissues, pathophysiological alterations impact typical cellular structures, resulting in variations in water diffusion. DWI can detect aberrant changes in diffusion and can be used to evaluate brain tissue’s integrity, connectivity, and structure (Martinez-Heras et al., 2021). Glaucoma is considered a multifactorial neurodegenerative disease affecting the visual pathway rather than just an ophthalmic condition with VF abnormalities and optic neuropathy. DTI, which was first introduced by Basser et al. (1994) in 1994, is a magnetic resonance technique based on DWI technology that can analyze the water molecule dispersion characteristics in tissue in three dimensions at regular intervals and quantitatively and has significant applications in neuroimaging. DTI enables a sensitive evaluation of potential microstructural changes that may occur prior to brain tissue shrinkage. Therefore, this technique is up-and-coming for the study of intracerebral damage in glaucoma (Le Bihan and Iima, 2015). DTI measures several parameters, including fractional anisotropy (FA), mean diffusivity (MD), axial diffusivity (AD), and radial diffusivity (RD). FA measures the propensity of water to diffuse in one direction, with values ranging from 0 (isotropic) to 1 (anisotropic) (Li and Zhang, 2020). MD describes the average mobility of water molecules within a voxel. In contrast, AD measures diffusivity along the horizontal axes, and RD offers an average diffusion measurement along the two vertical axes (Altobelli et al., 2015). Changes in these variables may signify alterations in axonal integrity and demyelination. Recently, DTI has been extensively utilized to evaluate glaucoma-induced impairments to the intracranial visual system in humans and animals.

## 4. DTI in glaucoma *in vivo*

Changes in DTI parameters of the glaucomatous ON and beyond the ON are hot areas of current research (Sidek et al., 2014; Zhang Q. J. et al., 2015). The following section focuses on developing DTI investigations of the visual pathway in animal and human populations with glaucoma.

### 4.1. DTI in animal models of glaucoma

Diffusion tensor imaging has been examined extensively and thoroughly in glaucoma-affected animal models with altered visual pathways in experimental animal models. Numerous investigations on rat glaucoma models have documented an increase in MD and a decrease in FA of the ON and OT (Hui et al., 2007; Ho et al., 2015; Yang et al., 2018). Moreover, *in vivo* DTI was demonstrated to detect RGC axonal damage earlier than immunohistochemistry in the early stages of the disease in a mouse model of optic nerve crush-induced glaucoma (Zhang et al., 2011). DTI can therefore be utilized to detect FA of OR and OT and aid in the early identification of POAG. DTI was used to test the visual system integrity in five animal models of glaucoma (Colbert et al., 2021). The evaluation of three glaucoma models that are caused by genes and two that are induced by experiments. Significantly less FA and more RD were found in the visual pathways of DBA/2J mice and LTBP2-mutant cats; also, AD was slower in DBA/2J mice. Chronic high IOP is linked to less FA and more RD along the ON or OT, which suggests that microstructural integrity has been compromised. In addition, Graham et al. (2021) use DTI to describe measurements of structures associated with canine primary angle closure glaucoma (PACG) in the retina and visual pathways beyond the optic papilla. Quantitative measurements of the ON, optic chiasma (OC), OT, and LGN were taken in dogs with and without PACG. A tendency toward a disease-related decline in FA was seen for all structures evaluated. *In vivo* evaluations of axonal, myelin, and trans-synaptic degeneration in canine PACG can be done using DTI. Research on DTI is currently being done primarily in rat eyes, and future research on glaucoma in large animals, including dogs, pigs, and monkeys, will also be done.

### 4.2. DTI in humans with glaucoma

Glaucoma-related modifications to DTI parameters in the visual pathway constitute a significant research focus. Multiple studies have found that glaucoma patients had lower FA and higher MD on DTI (Chen et al., 2013; Murai et al., 2013). Moreover, studies have established that FA is a more sensitive and reliable glaucoma detection indicator than MD (Garaci et al., 2009; Chen et al., 2013). These works of literature focus on the various types of glaucoma and the associated changes in the intracranial visual and extra-visual pathways involved in glaucoma (Giorgio et al., 2018; Schmidt et al., 2018; Wang et al., 2018; Qu et al., 2019). It is well-known that VF examination and OCT measurement of the RNFL thickness are the accepted methods for diagnosing primary open angle glaucoma (POAG) and ocular hypertension (OHT). And Song et al. (2018) indicate that DTI distinguishes POAG from OHT by evaluating the FA and MD of the OT, LGN, and OR in the visual pathway. DTI characteristics can quantify the evolution of POAG.

Schmidt et al. (2018) studied the potential advantages of volumetric LGN and DTI evaluation techniques. Normal tension glaucoma (NTG) was observed to considerably diminish the size of the LGN compared to healthy controls. In addition, FA of the OT and OR are lowered in NTG. Although RNFL thickness was related to LGN volume, FA was not correlated with LGN volume. Likewise, Li et al. (2019) examined the diagnostic value of DTI parameters and LGN size in POAG. FA values of the OT may be a sensitive and accurate biomarker for glaucoma assessment, even though MD is not connected with this condition. Zhou et al. (2017) discovered that the DTI parameters FA and RD correspond with monocular right and left visual fields, although there is no significant correlation between FA and RNFL thickness. The connection between FA and contralateral VF scores for OR is significantly positive. By splitting the left and right VF, FA readings for OR can be used to evaluate the degree of glaucomatous visual field loss. The results of the OT evaluation revealed no correlation. The results of the OT evaluation revealed no correlation. The OT is relatively narrow, has fewer neuronal bundles, and has poorer sensitivity, which may cause the absence of association. Engelhorn et al. (2011) used DTI to assess the pathological abnormalities in glaucoma patients and found that it can demonstrate the sparsity of OR. The study included 50 glaucoma patients and 50 age-matched healthy controls. Twenty-two glaucoma patients (44%) were found to have significantly lower OR volumes than the control group (67.16%). As a result, glaucomatous ON atrophy and OR thinning brought on by DTI coexist. Furthermore, compared to controls, glaucoma patients had significantly higher incidences of cerebral microangiopathy affecting OR.

Researchers are particularly interested in determining whether abnormalities occur outside the visual pathway in glaucoma patients. In contrast to nature controls, NTG patients show decreased FA of OR and forceps major in the occipital lobe, according to a DTI study by Boucard et al. (2016). The same changes were observed in non-visual regions, including the corpus callosum and parietal lobe. Moreover, Giorgio et al. (2018) discovered aberrant WM in the lingual gyrus and lateral occipital cortex of the occipital lobe in the NTG compared to the NC group, demonstrating a decrease in FA and an increase in AD. Moreover, Zikou et al. (2012) used DTI to identify decreases in FA in the inferior frontal-occipital fasciculus, the longitudinal and inferior frontal fasciculi, the putamen, the caudate nucleus, the anterior and posterior thalamic radiations, and the anterior and posterior limbs of the internal capsule.

As a result, we can now consider glaucoma a neurodegenerative disorder affecting the visual and extra-visual pathways owing to analyzing various types of glaucoma utilizing DTI. Additionally, DTI offers objective and quantifiable changes in the visual pathway and improves understanding of the central visual pathway’s degenerative process in human and animal glaucoma.

## 5. Correlation of DTI parameters and glaucoma severity

Multiple investigations have demonstrated that as the disease progresses, FA and MD values in the ON and OR decrease and increase, respectively (Garaci et al., 2009; Chen et al., 2013; Li et al., 2014). In addition, Sidek et al. (2014) found substantial variations between mild and severe glaucoma in the FA and MD of the ON and OR. FA values of either the ON or OR showed greater sensitivity and specificity in differentiating between mild and severe glaucoma than MD values. Similarly, Li et al. (2014) found that the heterogeneity analysis shows that FA may have a link with the severity of glaucoma. This study suggests that DTI may be helpful for glaucoma diagnosis and management. A meta-analysis investigated the connection between structural WM alterations and glaucoma severity. Typically, the severity of glaucoma increases the prominence of cerebral WM loss (Sidek et al., 2014). Therefore, based on the extent of parameter measurement in the visual pathway, DTI can differentiate the severity of glaucoma and offer some clinical support.

## 6. Limitations of DTI in glaucoma research

Application DTI has some limitations. The human ON is small, only 3–4 mm in diameter. There may be specific areas throughout the inspection when artifacts lead to examination errors (Wheeler-Kingshott et al., 2006). This restriction can be removed by utilizing advanced DTI and higher MRI scanners. It is important to remember that DTI parameters are voxel-based values that the fiber image located around the same voxel can change. Significant noise is detected for the DTI image, and the noise image leads to measurement inaccuracies.

Moreover, the DTI signal acquisition time is extended, which could cause motion distortions if the patient moves a lot during the examination. DTI should be enhanced and modified prior to its widespread usage in clinical glaucoma testing, which is now employed primarily for research. The accuracy and reproducibility of DTI results can be impacted by oculomotor interference, large magnetic sensitivity differences in the orbit caused by gas and bone in the sinus combined with the thin diameter of the optic nerve, encapsulation by cerebrospinal fluid in the sphincter cavity, and surrounding orbital fat (Techavipoo et al., 2009). The final results may need to be interpreted as a result of inaccurate diffusion measurements that need to adequately reflect microstructure information due to methodological and artificial factors (Concha, 2014). However, DTI measurements need a more precise biological meaning, and the method has many technical limitations. The DTI-based analysis is still an impenetrable technology that relies on complex data gathering and geometrical models that are predicated on many different assumptions (Pujol et al., 2015).

In this review, we only discuss the alterations of DTI in the visual and extra-visual pathways because not only WM alterations but also gray matter alterations are present in the central visual pathways of glaucoma patients, and intracranial gray matter alterations are typically detected by functional MRI (Garaci et al., 2008; Zhang et al., 2015), which is not discussed in this paper and is its limitation. Future research will delve deeper into glaucoma sufferers’ gray matter changes.

## 7. Future prospects

The area of glaucoma and ophthalmology, in general, is primarily image-based, and AI is situated to solve many of these problems (Mayro et al., 2020). Deep learning (DL) is a subset of AI. Using multilayer neural networks modeled after the mammalian visual cortex, DL in AI may generate images that modify the glaucoma field. Autonomous DL algorithms can maximize information in digital fundus pictures, optical coherence tomography, and visual fields (Ting et al., 2019; Girard and Schmetterer, 2020). Suppose DL technology can be merged with DTI to evaluate changes in images and parameters. In that case, it will be possible to explore the characteristics of glaucoma as a neurodegenerative disease and distinguish glaucoma patients from those without the problem. In the future, the combination of AI and DTI will significantly impact outpatient glaucoma screening, management, and the exploration of glaucoma’s relationship to other neurodegenerative illnesses. In addition, as technology improves and is combined with other MRI technologies (Lanzafame et al., 2016; Wang et al., 2018), glaucoma will be detected earlier and can be studied as a whole to find out how glaucoma starts, cease it from getting worse, improve the prognosis of glaucoma, and take glaucoma research to a new level, all of which will help glaucoma patients in the long run.

## 8. Conclusion

In summary, DTI often showed a rise in MD and a decrease in FA, strongly linked with increasing disease severity in glaucoma patients or animal models. DTI is a promising non-invasive technique for assessing the severity and prognosis of glaucoma. As clinical and scientific uses of DTI continue to develop, practitioners in the area will engage with ophthalmologists to overcome its limits and enhance patient care.

## Author contributions

All authors contributed to draft and revise the manuscript, gave final approval of the version to be published, and agreed to be accountable for all aspects of the work.
